# Immunogenicity Assessment of a 14-Valent Human Papillomavirus Vaccine Candidate in Mice

**DOI:** 10.3390/vaccines12111262

**Published:** 2024-11-08

**Authors:** Lei Bei, Shuman Gao, Dandan Zhao, Yajuan Kou, Siyu Liang, Yurong Wu, Xiao Zhang, Dan Meng, Jianbo Lu, Chunxia Luo, Xuefeng Li, Yang Wang, Hongbin Qiu, Liangzhi Xie

**Affiliations:** 1 Heilongjiang Pharmaceutical Research Institute, Jiamusi University, Jiamusi 154007, China; xiaoming68250@163.com (L.B.); qhbin63@163.com (H.Q.); 2 Beijing Engineering Research Center of Protein and Antibody, Sinocelltech Ltd., Beijing 100176, China; shuman_gao@163.com (S.G.);; 3 Beijing Key Laboratory of Monoclonal Antibody Research and Development, Sino Biological Inc., Beijing 100176, China; 4 Cell Culture Engineering Center, Chinese Academy of Medical Sciences & Peking Union Medical College, Beijing 100005, China

**Keywords:** HPV, vaccine, pseudovirion-based neutralization assay (PBNA), total IgG Luminex immunoassay (total IgG LIA), immunogenicity, correlation analysis

## Abstract

Background: Cervical cancer ranks as the fourth most common cancer affecting women globally, with HPV as the primary etiology agent. Prophylactic HPV vaccines have substantially reduced the incidence of cervical cancer. Methods: This study assessed the immunogenicity of SCT1000, a 14-valent recombinant virus-like particle (VLP) vaccine developed by Sinocelltech, Ltd. using pseudovirion-based neutralization assays (PBNAs) and total IgG Luminex immunoassays (LIAs). Currently in phase III clinical trials in China, SCT1000 targets the same HPV types as Gardasil 9^®^, plus five additional high-risk types, thereby covering twelve high-risk HPV types implicated in 96.4% of cervical cancer cases. Results: In murine models, a dose of 1.85 μg per mouse was identified as optimal for evaluating SCT1000’s immunogenicity in a three-dose regimen, as measured by PBNA and total IgG LIA across all 14 HPV types. SCT1000 induced high levels of protective antibodies, which were sustained for at least four months following the third dose. The vaccine also demonstrated stable and consistent immunogenicity in mouse potency assays under both long-term and accelerated conditions. Additionally, our studies revealed a strong correlation between the two serological tests used. Conclusions: SCT1000 elicited robust, durable, and consistent humoral immune responses across all 14 HPV types, indicating its potential as a broad-spectrum vaccine candidate against HPV types 6/11/16/18/31/33/35/39/45/51/52/56/58/59. The significant correlations observed between PBNA and total IgG LIA support the use of the Luminex-based total IgG method as a reliable and effective alternative for immunogenicity assessment in preclinical and future clinical vaccine development.

## 1. Introduction

Human papillomavirus is a common DNA virus that primarily infects epithelial and mucosal tissues, leading to both benign and malignant lesions in reproductive and non-reproductive systems [1]. More than 200 HPV genotypes have been identified, with about 20 classified as high-risk due to their cancer-causing potential. These strains can be categorized into high-risk and low-risk groups based on their oncogenic capacity. HPV16 and HPV18 are the most oncogenic high-risk types, responsible for approximately 70% of cervical cancer cases, as well as contributing to anal, genital, and oropharyngeal cancers, and they may even infect the middle ear mucosa [2,3,4,5]. Meanwhile, low-risk types such as HPV6 and 11 cause benign conditions like genital warts, which can significantly impact quality of life. Additionally, low-risk HPV has been associated with cervical cancer [4]. Cervical cancer ranks as the fourth most common malignancy among women worldwide [6,7]. HPV’s role in its development was first identified in the 1980s [8,9,10,11], leading to the development of preventive vaccines aimed at reducing the incidence of HPV-related cancers, including those of the cervix, anus, and oral cavity [2,12]. Screening, especially for at-risk populations, is recommended to further prevent HPV-related cancers [13].

Currently, there are six preventive HPV vaccines with different valences available on the market, each targeting specific HPV strains. These include the bivalent HPV16/18 vaccine Cervarix^®^ (GlaxoSmithKline Biologicals, London, UK, approved in the USA in 2007), the quadrivalent HPV6/11/16/18 vaccine Gardasil^®^ (Merck & Co., Inc., Rahway, NJ, USA, approved in the USA in 2006), the nonavalent HPV6/11/16/18/31/33/45/52/58 vaccine Gardasil 9^®^ (Merck & Co., located in Rahway, NJ, USA, approved in the USA in 2014), the bivalent HPV16/18 vaccine Cecolin^®^ (Xiamen Innovax Biotechnology, Xiamen, China, approved in China in 2020), the bivalent HPV16/18 vaccine Walrinvax^®^ [Pichia pastoris] (Shanghai Zerun Biotechnology (a subsidiary of Walwax Biotechnology), Shanghai, China, approved in China in 2022), and the newly approved quadrivalent HPV6/11/16/18 vaccine Cervavac^®^ (Serum Institute of India, Pune, India, approved in India in 2022) [14,15,16]. These licensed vaccines are based on the virus-like particles (VLPs) of the HPV L1 protein and have demonstrated high efficacy and immunogenicity when administered prior to HPV exposure [14,15]. Notably, Gardasil 9^®^ significantly extends cervical cancer protection from approximately 70% to 90% compared to HPV16/18-only vaccines [17].

HPV vaccination evokes immune responses that are significantly stronger than those observed with natural HPV infection [18]. Neutralizing antibodies play a critical role in HPV-vaccine-induced protection. Therefore, the World Health Organization (WHO) recommends immunogenicity as a surrogate clinical endpoint for HPV vaccine efficacy assessment, but the final decision is made by national regulatory authorities, which may include immunogenicity, persistent infection, or early-stage disease (such as cervical intraepithelial neoplasia 2 or higher) as endpoints [19]. Reports show that the Gardasil^®^, Cervarix^®^, Gardasil 9^®^, Cecolin^®^, and Walrinvax^®^ vaccines all provide strong and sustained protection against the HPV types covered by the vaccines and the associated diseases [14,20,21,22,23,24,25].

The evaluation of type-specific humoral immune responses following HPV vaccination involves detecting antibodies via neutralizing or binding assays and measuring the antibody binding strength (avidity) [26]. The primary serological assays that are used to assess antibody responses in clinical trials include pseudovirion-based neutralization assays (PBNAs), neutralizing monoclonal antibody competition immunoassays, and enzyme-linked immunosorbent assays (ELISAs) [27,28,29,30]. The PBNA is considered to be the “gold standard” for measuring HPV-vaccine-induced antibody activity [31]; however, its technical complexity, labor intensity, variability, and high cost limit its use in large-scale clinical trials (Table 1).

The competitive Luminex immunoassay (cLIA) evaluates neutralizing antibodies against a single conformational epitope of each HPV type [32]. In contrast, total IgG assays, such as the total IgG Luminex immunoassays (total IgG LIA) and standard ELISAs, measure all the IgG antibodies that directly bind to HPV VLPs [32]. These assays are noted for their sensitivity, reproducibility, simplicity, time efficiency, and high-throughput capabilities (Table 1). Numerous studies have directly compared various serological assays [29,33,34,35].

The immunogenicity correlations between the direct ELISA or single-epitope-based inhibition ELISA and PBNA for HPV16 and HPV18 antibody responses following Cervarix^®^ vaccination have demonstrated that direct ELISA data exhibit a stronger correlation with the PBNA, suggesting its potential as a surrogate for neutralizing antibody assessments [29]. Clinical trials of Gardasil^®^ have also demonstrated high correlations between the PBNA, cLIA, and total IgG LIA for detecting anti-HPV16 and 18 antibodies [33,34]. Additionally, studies using cynomolgus monkeys demonstrated a strong correlation between the PBNA and Luminex-based total IgG assay results for a 14-valent VLP-based vaccine (HPV 6/11/16/18/31/33/35/39/45/51/52/56/58/59) [35].

In this study, we evaluated the immunogenicity of a recombinant VLP vaccine (designated as SCT1000) in a mouse model using two serologic methods (the PBNA and total IgG LIA). Additionally, we assessed the stability of three batches of a 14-valent HPV vaccine candidate stored at 2–8 °C for 0, 3, 6, 9, 12, 18, 24, and 38 months; at 25 °C for 0, 12, and 24 weeks; and at 37 °C for 0, 4, and 12 weeks in a mouse potency assay. In addition, we analyzed the correlation between the data obtained from the PBNA and total IgG LIA. SCT1000, developed by Sinocelltech, Ltd. (Beijing, China), is currently undergoing a phase III clinical trial in China (clinical trial number: CTR20232472). It not only targets the same HPV types as the 9-valent HPV vaccine Gardasil 9^®^ (HPV 6/11/16/18/31/33/45/52/58) but also includes five additional high-risk oncogenic types designated by the WHO (HPV 35/39/51/56/59). The 14-valent vaccine has the potential to prevent more than 96.4% of cervical cancers theoretically [17], as well as other HPV-related cancers.

## 2. Materials and Methods

### 2.1. HPV VLP Preparation

This recombinant 14-valent VLP vaccine (code-named SCT1000) covers 5 more high-risk oncogenic HPV types (HPV 35/39/51/56/59) listed by the WHO than the previously approved Gardasil 9^®^ (HPV 6/11/16/18/31/33/45/52/58).

The HPV VLPs used in this study were manufactured by Sinocelltech Ltd. The production expression system used the Bac-to-Bac^®^ insect virus expression system. For each HPV type, recombinant baculovirus was generated by constructing a plasmid containing the corresponding L1 gene and then transfecting the baculovirus into SF9 insect cells (ThermoFisher, Waltham, MA, USA). In these SF9 cells, the L1 proteins were produced as a result of baculovirus infection and subsequently self-assembled into VLPs of each specific HPV type. The recombinant baculovirus carrying the HPV-L1 gene was used to infect HighFive [36] insect cell line (ThermoFisher, Waltham, MA, USA) to promote the production of VLP protein. After the culture process, the cells were harvested by centrifugation. The collected cell pellet was lysed, and the resulting supernatant was collected after further centrifugation. The collected supernatant was subjected to a series of purification steps, including chromatography, denaturation, virus inactivation and removal processes, reaggregation, and ultrafiltration to prepare the drug substance. The quality confirmation was described in Supplementary Material S1.1.

### 2.2. Vaccine Formulation

Each batch of the HPV VLP drug substance is absorbed on an aluminum phosphate adjuvant. Subsequently, the VLPs of each HPV type absorbed with the adjuvant are mixed in a predetermined ratio (as shown in Table 2) to produce a semi-finished product. This semi-finished product is then filled and packaged to obtain the final vaccine formulation, which is called the SCT1000 drug product, a 14-valent HPV vaccine candidate.

### 2.3. Animal Immunization, Sampling, and Serological Analysis

All animals were housed and maintained in facilities accredited by the Association for the Assessment and Accreditation of Laboratory Animal Care (AAALAC), ensuring compliance with rigious standards of animal welfare throughout the study. All experimental procedures were conducted in accordance with Chinese animal use guidelines, approved by the Institutional Animal Care and Use Committee (IACUC), and adhered to the ARRIVE guidelines, as endorsed by Multidisciplinary Digital Publishing Institute (MDPI), to ensure ethical animal treatment and transparent reporting (Supplementary Material S1.2).

To evaluate the immunogenicity of SCT1000, the 14-valent vaccine candidate was diluted to 0.5-fold (0.5×), 1-fold (1×), 1.25-fold (1.25×), and 2-fold (2×). Female specific pathogen-free (SPF) Balb/c mice aged 6–8 weeks were immunized by subcutaneous injection of 200 μL of SCT1000 diluted in aluminum phosphate adjuvant. Serum samples were collected according to the prime-boost immunization schedule and sampling time points (Table 3 and Table S1) and analyzed for neutralization titers and ED_50_ (the half effective dose) values using PBNA and total IgG using total IgG LIA. All mice used in this study were 6- to 8-week-old female mice and were randomized into groups.

In Study #1, two assays were performed. In the first assay, a group of 40 6- to 8-week-old female SPF Balb/c mice were vaccinated with four dilutions of vaccine samples—0.5× (0.93 μg/mouse), 1× (1.85 μg/mouse), 1.25× (2.31 μg/mouse), and 2× (3.70 μg/mouse)—on days 0, 7, and 21. In the second assay, 20 female SPF Balb/c mice of the same age range were vaccinated with two dilutions of vaccine samples—1× (1.85 μg/mouse) and 1.25× (2.31 μg/mouse), following the same schedule. Each dilution was administered to ten mice. Serum samples were collected on day 28. All serum samples were then inactivated at 56 °C for 30 min and subsequently analyzed for pseudovirion-based neutralization titers and Luminex-based total IgG.

In Study #2, four assays were conducted, with each assay comprising groups of 10, 30, 10, and 10 animals, respectively. One of the groups was obtained from assay 1 in Study #1. A total of 60 female SPF Balb/c mice, aged 6 to 8 weeks, were immunized subcutaneously with the 1-fold diluted vaccine samples (1.85 μg/mouse) on days 0, 7, and 21. Serum samples were collected on day 28, one week after the third vaccination. All serum samples were heat-inactivated at 56 °C for 30 min and subsequently analyzed for pseudovirion-based neutralization titers and Luminex-based total IgG.

In Study #3, 51 6- to 8-week-old female SPF Balb/c mice from five groups were also vaccinated with the 1-fold diluted vaccine samples (1.85 μg/mouse) on days 0, 7, and 21. Serum samples were collected on day 28 (one week after dose 3), 35 (two weeks after dose 3), day 56 (five weeks after dose 3), 63 (six weeks after the third dose), and 105 (twelve weeks after dose 3). Of note, 10 of the serum samples collected on day 28 were from assay 3 of Study #1. Unfortunately, one mouse was lost on day 105. All serum samples were subsequently inactivated at 56 °C for 30 min and analyzed for pseudovirion-based neutralization titers and Luminex-based total IgG.

Mouse potency testing was based on previous studies [37]. For this assay, seven three-fold dilutions were prepared for each 14-valent HPV vaccine sample tested. The immunizing dose range for HPV31, 33, 35, 39, 45, 51, 52, 56, 58, and 59 was 0.00014–0.1 μg/mouse, and the doses for HPV6, 11, 16, and 18 were adjusted proportionally according to their respective proportions in the final product (Table 2 and Table S2). Further, 6- to 8-week-old female Balb/c mice (n = 10 per dosage) were vaccinated with seven doses of aluminum-phosphate-adjuvanted 14-valent vaccine. Groups with 10 unimmunized mice and 10 adjuvant-immunized mice were also set up.

Mouse sera were collected on day 28. All sera samples were then inactivated at 56 °C for 30 min and then tested in PBNA. The percentage of mice that seroconverted was calculated for each dose. The reportable result is the theoretical dose that would induce seroconversion in 50% of the animals in a given group (ED_50_). Mouse potency results are reported in micrograms of protein per mouse. Mouse potency testing was also performed on 14-valent HPV production batches stored at 2~8 °C, 25 °C, and 37 °C (Table 4).

### 2.4. Preparation of HPV Pseudovirions

All 14 HPV pseudovirions were generated by co-transfecting 293 FT cells (Institute of Basic Medical Sciences, Chinese Academy of Medical Sciences) with expression plasmids encoding the HPV L1 and L2 capsid genes and a GFP (green fluorescent protein) reporter plasmid using Sinofection transfection reagent (STF02, Sino Biological Inc., Beijing, China). HPV L1-L2 genes were constructed in pCMV3 plasmid, while GFP gene was in pCDNA3.1 plasmid. After transfection, cells were harvested 48 h after transfection and lysed with 10% Triton X-100, Super Nuclease (SSNP01, Sino Biological Inc., Beijing, China), Plasmid-Safe ATP-dependent DNase (E3101K, Lucigen Corporation, Middleton, WI, USA), and 1 M ammonium sulfate. The cell lysate was then incubated overnight at 37 °C to allow the maturation and extraction of the produced pseudovirions, followed by transfer of 0.85 M NaCl solution and clarification by centrifugation at 10,000× *g* for 10 min. The GFP expression levels of the 14 different pseudovirus types were assessed using the ImmunoSpot Analyzer (Cellular Technology Ltd. (CTL), Cleveland, OH, USA). The measured GFP signal for each pseudovirus type was adjusted to fall within a predetermined and validated range of GFP signal spots, as described in Table S3.

To titrate pseudovirions, 293 FT cells were pre-seeded into 96-well plates containing 100 μL of cell culture medium at a density of 15,000 cells per well. After incubation at 37 °C for 3–6 h, the pseudovirions were serially diluted two-fold and infected into 293 FT cells. After incubation at 37 °C, with 5% CO_2_ for 68–96 h, fluorescent spots were counted using CTL. An optimal dilution of approximately 400 fluorescent spots is recommended for use in subsequent validation procedures.

### 2.5. PBNA

**Figure 1:**
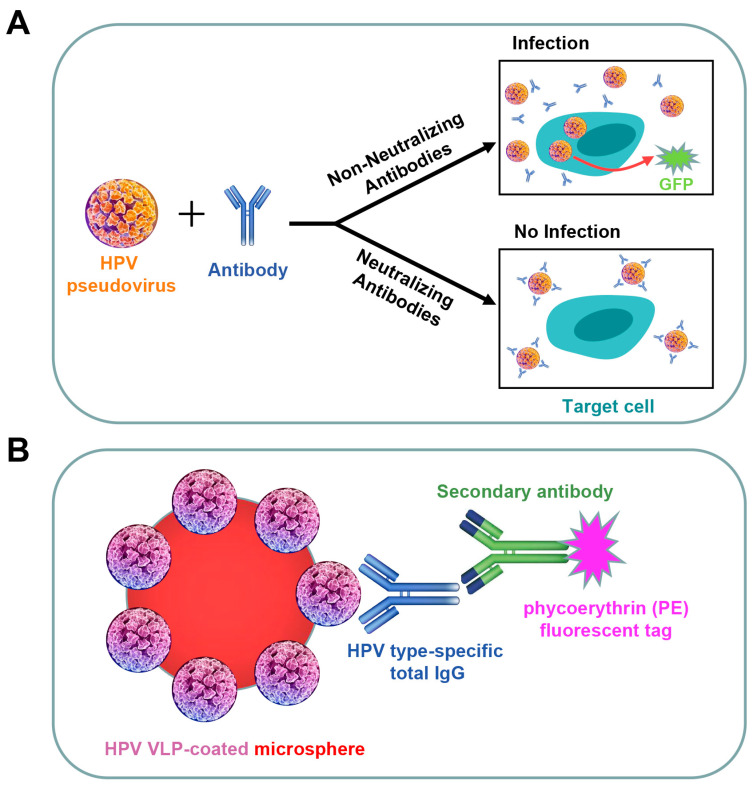
Principles of PBNA and total IgG LIA. (**A**). Illustration of the cell-based PBNA. Neutralizing antibodies block GFP expression in target cells by binding to pseudoviruses and inhibiting pseudovirus infection. Non-neutralizing antibodies do not block infection and GFP expression. The increased presence of type-specific neutralizing antibodies results in a decrease in the number of cells expressing GFP, which can be quantified using the ImmunoSpot Analyzer (CTL). (**B**). Luminex-based total IgG LIA principle. The assay uses HPV VLPs coupled to fluorescent Luminex magnetic beads to quantify type-specific antibodies in mouse sera. Antibodies bound to type-specific epitopes on the VLPs are detected using a PE-labeled anti-mouse secondary antibody. The fluorescence signal value (MFI) positively correlates with the level of total type-specific IgG in the serum.

HPV PBNA used in this study is a modified version of a previous method [38] and was developed by Sinocelltech Ltd. [35]. The mechanism of PBNA is shown in Figure 1A. PBNA has been fully validated, as described in Supplementary Material S1.3.

Mouse sera were centrifuged at 10,000× *g* for 5 min, and 293 FT cells were pre-seeded in 96-well flat bottom cell culture plates at 15,000 cells/well, and 100 μL of cell culture medium was added. The culture plates were then incubated at 37 °C for 4–8 h. Mouse sera were serially diluted and mixed separately with 14 diluted pseudovirion stocks. After incubation at 4 °C for 1 h, 100 μL of the pseudovirion–serum mixture was transferred to the pre-seeded 293 FT cells and incubated at 37 °C for 60–96 h. Each sample was tested in two wells. Additionally, control wells containing only pseudovirions, only culture medium, and pseudovirion–blank mouse serum mixtures were set up as positive, negative, and blank serum controls, respectively. The GFP signal was quantified using an immunospot analyzer (CTL), and the neutralization rate of each sample was calculated according to the formula.

(1) $$\mathrm{Neutralization}\;(\%)=\;(1-\frac{\mathrm{Immune}\;\mathrm{sera}-{\mathrm{Background}}_{\mathrm{No}\;\mathrm{pseudovirion}}}{\;\mathrm{Blank}\;\mathrm{mice}\;\mathrm{control}\;\mathrm{sera}-{\mathrm{Background}}_{\mathrm{No}\;\mathrm{pseudovirion}}})\times 100\%$$

The neutralization titers were calculated using the Reed–Muench method and represented the highest dilution capable of inhibiting 50% green fluorescent protein (GFP) expression (EC_50_).

To determine the cutoff value for the PBNA method and eliminate nonspecific reactions from mouse serum background, blank mice serum pools from three different sources were used. Neutralization titer testing showed that the background signal of mouse serum was relatively small when diluted to 1:80 or above.

### 2.6. Preparation of VLP-Coated Magnetic Beads

The preparation of 14 performed VLP-coated magnetic beads included two steps. In the first step, the carboxyl groups on the surface of the microspheres (Luminex Corporation, Austin, TX, USA) were activated with 1-ethyl-3-(3-dimethylaminopropyl)-carbodiimide hydrochloride (EDC) to form N-hydroxy-sulfosuccinimide (NHS)-ester transition products in the presence of NHS. In the second step, covalent amide bonds were formed between the transition products and the primary amines of the protein. The concentrations of EDC and NHS were adjusted according to the different types, maintaining a ratio of 2.5 × 10^6^ magnetic beads to 5 μg of protein. The coupled beads were counted with a cell counter and then diluted to 2 × 10^6^ beads/mL with preservative buffer and stored at 2~8 °C in the dark. HPV VLP drug substances were used as coating antigens and VLPs were prepared and purified according to the steps outlined in the Section 2.1. These VLPs showed good specificity, and the establishment of total IgG LIA using VLP-conjugated magnetic beads based on the Luminex platform ensured high precision and sensitivity.

### 2.7. Total IgG LIA

The in-house development of the total IgG LIA involved multiple optimizations based on previously published studies [32,35]. The underlying mechanism of the Luminex-based total IgG LIA is shown in Figure 1B. The core of Luminex technology is to adjust the ratio of fluorescent dyes to encode multiple magnetic beads, each representing a different fluorescence spectral feature. Further, 14 commercial magnetic beads were covalently cross-linked to HPV VLPs, which are HPV VLP drug substances produced for vaccines. The resulting VLP-coupled magnetic beads were then incubated with mouse serum samples. Detection was performed using an anti-mouse specific antibody labeled with phycoerythrin (PE) and then analyzed using the Luminex 200 instrument (Luminex Corporation, Austin, TX, USA). It has been successfully extensively validated using a cynomolgus monkey model [35] and a human clinical trial serum matrix [39] (Supplementary Material S1.4).

In the assay, 50 μL of PBST containing 1% BSA was added to each well of a blank 96-well plate. The plate was then washed at room temperature for at least 30 min, after which the supernatant was removed. Mouse serum was centrifuged at 6000× *g* for 5 min, the resulting supernatant was diluted 5000-fold, and 25 μL per well was added to the 96-well plate. Additionally, 25 μL of the pre-prepared VLP-coated beads of each of the 14 types was mixed and added to the 96-well plate (equating to approximately 2000 VLP-coated beads per well for each HPV type). The plate was incubated at room temperature in the dark for 2 h.

Subsequently, the plate was moved to a plate washer for washing. The secondary antibody used for detection was Invitrogen’s goat anti-mouse IgG(H + L) cross-adsorbed phycoerythrin (PE) labeled secondary antibody. The secondary antibody was diluted 400 times, 50 μL of diluents was added to each well, and the plates were incubated at room temperature in the dark for about 1 h. The plates were moved to a plate washer for washing, 100 μL of sample diluent was added to each well to resuspend the magnetic beads, and the samples were analyzed using the Luminex system.

To mitigate potential interference from mouse serum background, cutoff values for the total IgG LIA method were determined. To this end, serum samples were collected from more than 100 naïve mice and used to evaluate the cutoff points for the total IgG LIA method. Cutoff values for each HPV type were calculated using the mean + 3.09 SD method, with a target of 0.1% false-positive rate based on negative sera response (MFI). Detailed results of these calculations are listed in Table 5.

### 2.8. Statistics

Neutralizing antibody titers and MFI readings were calculated as geometric mean titer (GMT) ± 95% confidence interval (CI) or standard error (SE). ED_50_ was calculated as mean ± SEM. Pearson correlation analysis (*p* values, two-tailed) was performed separately for each HPV type. These calculations were performed using GraphPad Prism software Version 8.0.1 and Excel. A significance threshold of *p* < 0.05 was used to indicate a significant correlation between the PBNA and total IgG LIA data.

## 3. Results

### 3.1. Optimal Immunization Dosage of a 14-Valent Vaccine Candidate

**Figure 2:**
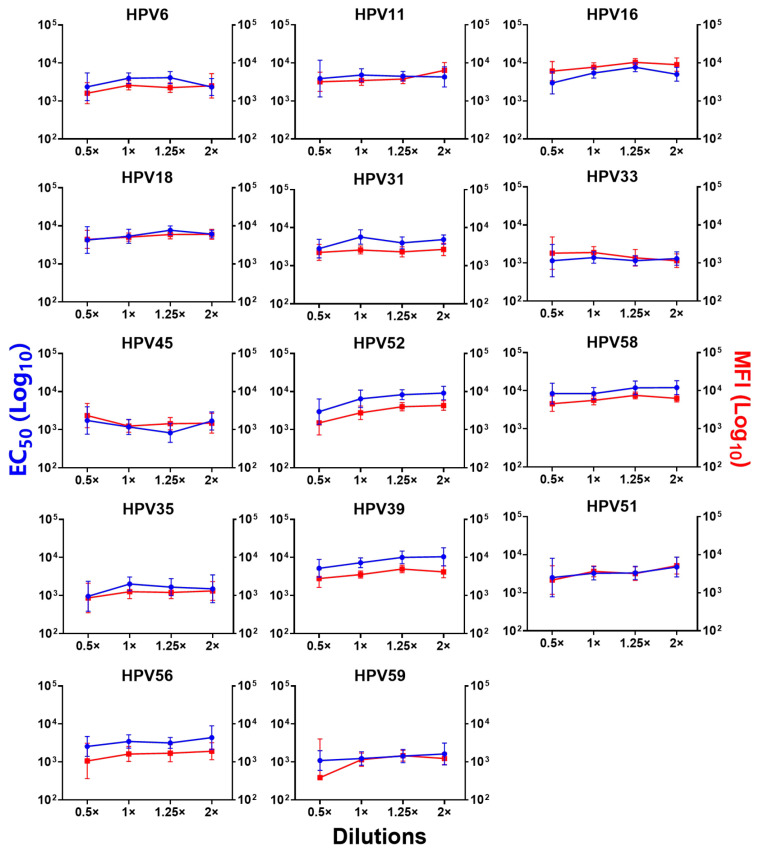
Immunogenicity analysis of a 14-valent HPV candidate vaccine administered to BALB/c mice at different dosages in Study #1. In a dose escalation study (Study #1), geometric mean titers (GMTs) of 14 HPV types were measured using PBNA and total IgG LIA. The 0.5-, 1-, 1.25-, and 2-fold dilutions represent total HPV doses of 0.93 μg, 1.85 μg, 2.31 μg, and 3.70 μg per mouse for the 14-valent vaccine candidate, respectively. The solid black line and left vertical axis represent the PBNA titers, and the solid red triangles and right vertical axis represent total IgG LIA results (MFI). Error bars represent 95% confidence intervals (CIs) for GMTs.

To determine the optimal immunization dose of the 14-valent vaccine candidate in mice, we performed a dose-escalation study with a three-dose regimen (Study #1). The results showed that all the vaccine dose groups induced strong antibody responses against all 14 target HPV types. For most types, we found a gradual increase in the antibody responses at different immunization doses (from 0.93 to 1.85 μg per mouse) except for HPV11, 33, and 45, as assessed by the PBNA and total IgG LIA assays. The antibody response remained relatively stable within the range of 1.85 μg to 2.31 μg per mouse, after which the antibody response decreased, especially for HPV6 and HPV16, or increased for HPV39, 51, and 56 (Figure 2). Therefore, we selected 1.85 μg per mouse as the optimal dose for evaluating the immunogenicity of the 14-valent HPV vaccine candidate.

### 3.2. The 14-Valent HPV Vaccine Candidate Can Induce Strong and Long-Lasting Humoral Immune Response

**Figure 3:**
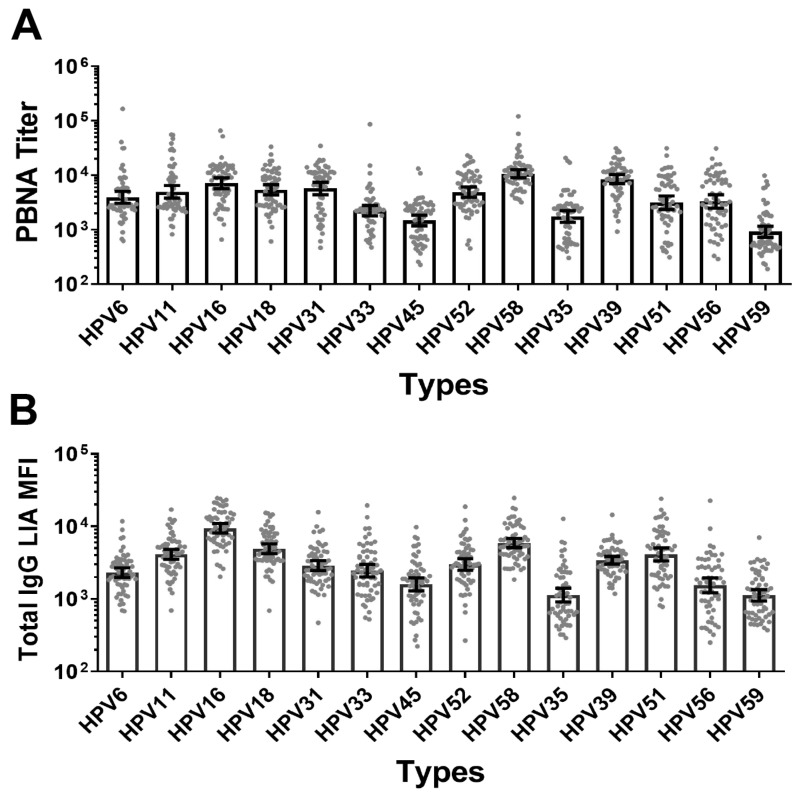
Immunogenicity analysis of a 14-valent HPV candidate vaccine administered to BALB/c mice at a single dosage in Study #2. A total of 60 mice were vaccinated with a 1-fold dilution (1.85 μg) of the candidate vaccine. Sera were tested with PBNA (**A**) and total IgG LIA (**B**). Geometric mean titers (GMTs) for 14 HPV types were measured. Error bars represent 95% confidence intervals (CIs) of GMTs.

To further evaluate the neutralization titers evoked by the 14-valent vaccine candidate in mice, four groups of Balb/c mice were vaccinated with a single dose of the 14-valent SCT1000 vaccine (1.85 μg/mouse) in a prime and boost schedule (Study #2). The results showed that the 14-valent SCT1000 vaccine at 1.85 μg/mouse induced a strong humoral immune response against the 14 HPV types, as measured by the PBNA and total IgG LIA (Figure 3 and Figure S4). The neutralizing antibody titers and total IgG levels varied among the HPV types. The GMT neutralization titers for the 14 HPV types ranged from 919 to 10,693, and the GMT MFI for the 14 HPV types ranged from 1121 to 9437.

**Figure 4:**
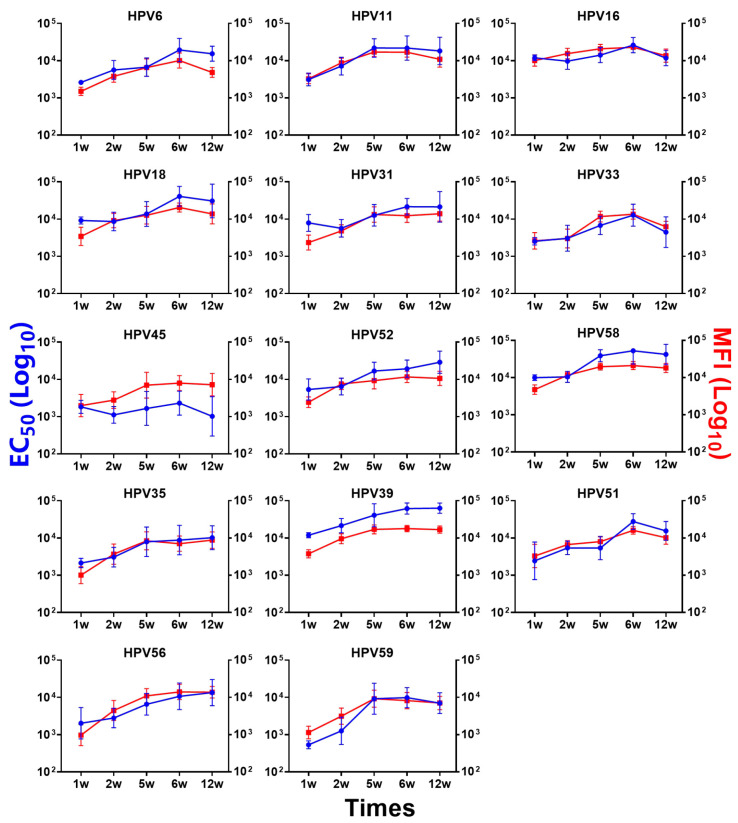
Immunogenicity analysis of a 14-valent HPV candidate vaccine administered to BALB/c mice in Study #3. In Study #3, geometric mean titers (GMTs) for 14 HPV types were measured by PBNA and total IgG LIA on days 28, 35, 56, 63, and 105. The solid blue lines and left vertical axis represent the PBNA results. The solid red squares and right vertical axis represent the total IgG LIA results. Error bars represent the 95% confidence intervals (CIs) of the GMT.

To clarify the persistence of immunogenicity of the 14-valent vaccine candidate in mice, we conducted a long-term immunization study in Study #3. The results showed that SCT1000 maintains high antibody levels in both serological methods (PBNA and total IgG LIA assays), and the results for each HPV type also showed similar trends. Specifically, for most types, the type-specific antibody responses gradually increased at 1, 2, 5, and 6 weeks after the third dose and reached peak antibody levels 6 weeks after the third dose (D63) (Figure 4). In mice, high protective antibody levels persisted for at least four months after the third dose (Figure 4).

### 3.3. The 14-Valent HPV Vaccine Shows Consistent and Stable Efficacy in Different Batches

**Figure 5:**
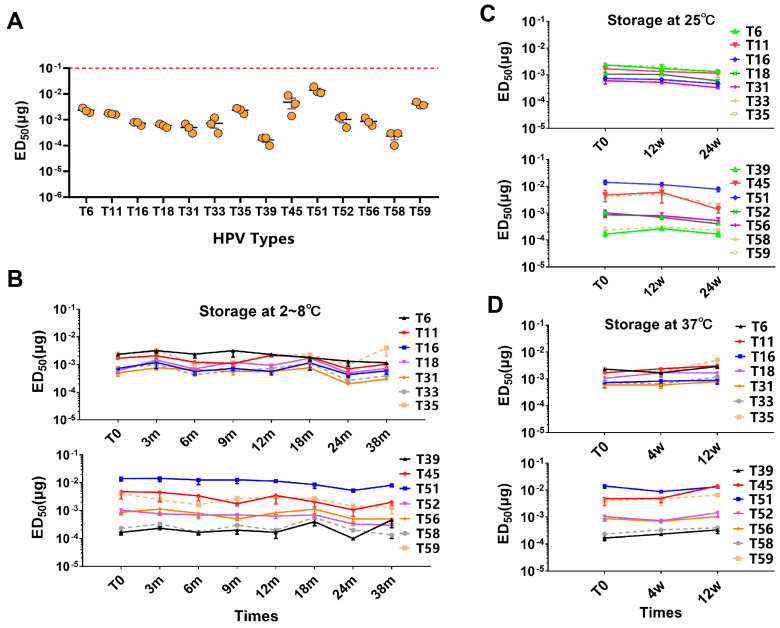
The consistency and stability of the 14-valent HPV vaccine candidate in a mouse potency assay. (**A**). Mouse efficacy of three batches of 14-valent HPV vaccine candidates stored at 2~8 °C. The vertical axis represents ED_50_ and standard error (SE) of the mean. (**B**). Mouse efficacy of three batches of 14-valent HPV vaccine candidates stored at 2~8 °C for 0, 3, 6, 9, 12, 18, 24, and 38 months. (**C**). Mouse efficacy of three batches of 14-valent HPV vaccine candidates stored at 25 °C for 0, 12, and 24 weeks. (**D**). Mouse efficacy of three batches of 14-valent HPV vaccine candidates stored at 25 °C for 0, 4, and 12 weeks.

We quantified the immune response of mice to SCT1000 using a median effective dose (ED_50_) assay. ED_50_ was defined as the minimum vaccine dose that induced seroconversion in 50% of the animals in a given group. Balb/c mice were vaccinated with seven dilutions of SCT1000. Serum was collected 4 weeks after immunization to measure the pseudovirion-based ED_50_. Using Reed–Muench statistical analysis, the calculated average ED_50_ for the 14 HPV types ranged from 0.2 to 14.9 ng, with variability among the types (Table S2). Specifically, the average ED_50_ values of HPV39, 58 and HPV51 were 0.2 ng and 14.1 ng, respectively, the lowest and highest values among the 14 HPV types (Table S2). The ED_50_ values of the three HPV production batches for each type were highly consistent at 2–8 °C (Figure 5A).

The ED_50_ of SCT1000 stored at 2–8 °C remained stable for at least 38 months (Figure 5B), indicating that long-term storage at 2–8 °C for up to 3 years had no effect on the mouse potency ED_50_. At 25 °C, the ED_50_ of each SCT1000 remained stable for at least 24 weeks (Figure 5C), and, at 37 °C, it remained stable for at least 12 weeks (Figure 5D). These results indicate that the mouse potency of the 14-valent HPV vaccine candidate remains stable under accelerated conditions. Therefore, the 14-valent HPV vaccine candidate has consistent and stable immunogenicity under long-term and accelerated conditions in mice.

### 3.4. Correlation and Consistency Between PBNA and Total IgG LIA in the Mouse Model

**Figure 6:**
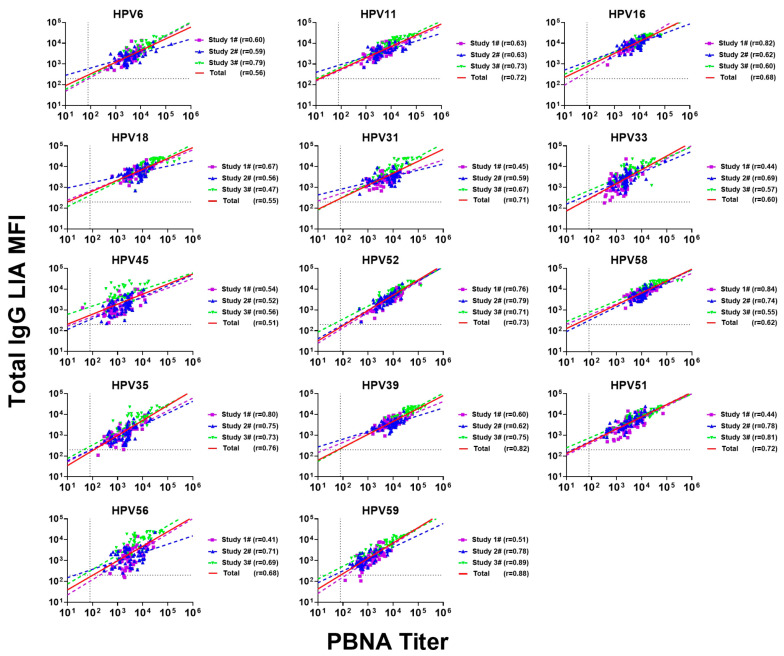
Correlation coefficients in individual studies and combined Study #1, Study #2, and Study #3. The figure shows the correlation between neutralizing antibody titers measured by PBNA and MFI readings measured by total IgG LIA when used alone and in combination in studies #1, #2, and #3. The horizontal axis represents the neutralizing antibody titers, and the vertical axis represents MFI readings. The horizontal and vertical dashed lines represent the cutoff values of total IgG LIA and PBNA for 14 HPV types, respectively. The slanted line represents linear regression. Pearson correlation coefficients (r values) are shown in the figure. Different symbols and colors represent data from different studies. Purple squares with purple regression lines represent data from Study #1, blue triangles with blue regression lines represent data from Study #2, green inverted triangles with green regression lines represent data from Study #3, and the red regression lines represent the combined or total data from all three studies (n = 150).

The total IgG LIA assays have several advantages over the PBNA, such as high throughput and reduced sample volume requirements (Table 1). The correlation between these two serological methods has been described in other papers published by us using human [39] and cynomolgus monkey [35] models. To further expend the application of this assay to mouse models, we analyzed the functional relationship between the PBNA and total IgG LIA assays for various HPV types in mouse models in three studies. In a dose-escalation study (Study #1), a statistically significant correlation was found between the neutralization titers (PBNA) and total IgG levels (total IgG LIA) (*p* < 0.001). The results showed that the correlation coefficients for HPV16, HPV35, and HPV58 were high (r > 0.80), while the correlation coefficients for HPV31, HPV33, HPV51, and HPV56 were relatively low (0.41 < r < 0.5) (Figure 6 and Table 6).

In the single-dose study (Study #2), there was a statistically significant (*p* < 0.0001) correlation between the two serological tests for all 14 HPV types. For the 14 HPV types, all the Pearson correlation coefficients ranged from 0.52 to 0.79, with HPV45 showing a relatively lower correlation (r = 0.52) (Figure 6 and Table 6). In a long-term study (Study #3), the results showed strong correlations (at least 0.55) for all the HPV types with the exception of HPV18 (r = 0.47)), which had the lowest correlations (Figure 6 and Table 6). The correlations remained highly significant, with *p* values less than 0.0001.

Combining the data sets of Study #1, Study #2, and Study #3 (n = 150), the overall analysis showed that the correlation coefficients ranged from 0.51 to 0.88, and all 14 HPV types showed significant correlations (*p* < 0.0001) (Figure 6 and Table 6). The correlations of HPV11, 31, 52, 35, 39, 51, and 59 were particularly significant, with correlation coefficients greater than 0.71. Among them, HPV59 had the highest correlation coefficient of 0.88 (Figure 6 and Table 6). Although the combined correlation coefficients and regression line slopes of the three studies were mostly consistent, there were some deviations, such as the low combined correlation for HPV6 and the high combined correlation of HPV31 and HPV39. The coefficients of variation (CVs) of the four analyses ranged from 4% to 27% (Table 6), indicating that different immunization schemes may introduce factors that affect correlation deviations. These results strengthen the reliability of the correlation between the MFI values obtained by the Luminex-based LTI method and the neutralization titers measured by the PBNA method in the mouse model. This demonstrates the versatility and reliability of this assay in multiple models, including mice, which is crucial for broader application in HPV vaccine development.

## 4. Discussion

Numerous studies have demonstrated the effectiveness of vaccines against human papillomavirus [40]. Gardasil 9^®^ is the vaccine with the broadest coverage that is currently on the market, and it has demonstrated strong protection against HPV6/11/16/18/31/33/45/52/58 infection and related cancer [36,41,42]. However, it does not provide protection against all high-risk HPV types. This underscores the urgent need to develop vaccines that offer broader coverage against additional high-risk HPV types. Therefore, there is an urgent requirement to develop HPV vaccines that cover more types. Humoral immunity has been recognized as a crucial factor in protection and in assessing the efficacy of HPV vaccines [43]. This study evaluated the immunogenicity and functional relevance of the investigational 14-valent HPV vaccine candidate, SCT1000, in a mouse model. The 14-valent SCT1000 expands the protection to include five additional high-risk HPV types (35/39/51/56/59) not covered by Gardasil 9^®^. Our results indicate that SCT1000 induces potent and durable humoral immune responses, demonstrating its potential as a comprehensive HPV vaccine.

### 4.1. Immunogenicity of 14-Valent HPV Vaccine Candidate

Several assays, such as the PBNA, total IgG LIA, and ELISA, have been developed to measure the antibodies evoked by HPV L1-VLP-based vaccines [27,28,29,30]. Here, we focused on evaluating the immunogenic potential of the 14-valent HPV vaccine candidate using these assays in a mouse model. Our results demonstrate that SCT1000 generates strong and durable humoral immune responses in this model, highlighting its potential as an effective multivalent HPV vaccine candidate. Furthermore, these findings highlight the value of using mice as a research tool in HPV vaccine development.

The main role of HPV vaccination is to provide primary prevention against HPV-related diseases [3,44]. The 14-valent HPV vaccine offers broader protection by not only reducing the risk of cervical cancer but also helping to prevent other HPV-related cancers. Gardasil 9 is indicated for the prevention of cervical, vulvar, vaginal, anal, oropharyngeal, and other head and neck cancers caused by HPV types 16, 18, 31, 33, 45, 52, and 58, as well as for genital warts (condyloma acuminata) caused by HPV types 6 and 11. We have compared the immunogenicity of Gardasil 9 with that of the 14-valent HPV vaccine in both preclinical and clinical trials (data to be published separately). The five additional HPV types covered by the 14-valent HPV vaccine may provide increased protection against HPV-related diseases compared to Gardasil 9. Although HPV vaccination is not recommended for the treatment of established HPV infections or anogenital warts, recent studies have suggested a potential therapeutic role for HPV vaccination in cases of genital warts that are resistant to standard therapies [44]. While the exact mechanism underlying this therapeutic effect remains unclear, it is thought that the vaccine may activate cellular immunity rather than humoral immunity [44]. The efficacy of the 14-valent HPV vaccine is primarily based on its ability to induce a strong humoral immune response, but its potential therapeutic applications warrant further investigation.

### 4.2. Correlation Between the Two Serological Assays

This is the first time that a strong correlation between a high-throughput Luminex-based total IgG method and a PBNA method has been demonstrated in a mouse model, which is widely used as a preclinical model for vaccine development. This finding highlights the feasibility and effectiveness of the Luminex-based total IgG method as a promising alternative for preclinical immunogenicity assessments, with potential applications in human clinical trial evaluations. This observed concordance is consistent with the published data from human clinical trials involving HPV types 16 and 18 [33,34] and the data on 14 HPV types in humans [39] or cynomolgus monkey models [35]. Our studies demonstrate a strong correlation between the two serological tests regardless of the serum source (from mice, as evaluated here, or from non-human primates, as reported by Bei et al., 2022) and the immunization schedule employed. The results from three studies (Study #1, Study #2, and Study #3) showed that, despite the small sample sizes (n = 50~60) and varying immunization protocols (multiple doses in Study #1 and single doses in Study #2 and Study #3), the two approaches exhibited consistent and comparable results. The median fluorescent intensity (MFI) values were positively correlated with the serum total IgG levels across a range of values. The CVs between the MFI-based and unit-based [35] correlation coefficients ranged from 0 to 6% (Table S5), indicating that MFI accurately reflects specific antibody concentrations and correlates well with pseudovirion-based neutralization titers. These results highlight the versatility and reliability of this assay in various host systems, extending its application to mouse models for the first time, which is crucial for its broader application in HPV vaccine development.

Our findings demonstrate that the total IgG LIA can effectively predict and capture neutralization responses detected by PBNAs across multiple models. However, although binding assays exhibit a strong correlation with neutralization assays, they cannot completely replace functional neutralization assays for assessing antibody potency as binding does not always equate to neutralizing activity. Therefore, binding assay results should be supplemented with functional assessments to obtain a comprehensive understanding of antibody function.

### 4.3. Limitations of These Results

The relatively small dose increments used in this experiment may have contributed to the small differences in the antibody responses observed among the dose groups. Subtle variations may mask more significant immune responses that might occur with larger dose changes. This limited range may explain the relatively stable antibody responses between 1.85 μg and 2.31 μg per mouse, highlighting the necessity to optimize the dose range in future studies. Although comprehensive method validation in mouse models was not feasible due to the limited sample size, our results suggest that this limitation does not compromise the robustness and accuracy of the Luminex-based total IgG method. Extensive validation in cynomolgus monkey models [35] and human clinical trial serum matrices [39] has consistently demonstrated the reliability and precision of the Luminex-based total IgG assay, highlighting its suitability for assessing immunogenicity across various serum matrices.

Another limitation that needs to be acknowledged is the potential impact of the host cell system on antibody reactivity and correlation analysis. The pseudovirions used in the PBNA were prepared in mammalian cells, while the VLPs used in the total IgG LIA were expressed in insect cells. This discrepancy in the cell systems may influence the detection of vaccine-specific epitopes, potentially leading to the generation of binding antibodies that could affect the correlation results. Further studies using VLPs prepared in mammalian expression systems are necessary to better understand and address the potential interference from vaccine-specific epitopes. In addition, high-titer vaccine responses that are primarily directed against specific vaccine epitopes often dominate the overall serological profile, which may obscure low-titer nonspecific reactions that are less correlated with neutralization assays. This limitation should be considered as they may impact correlation analysis.

## 5. Conclusions

In this study, a 14-valent HPV vaccine candidate exhibited robust immunogenicity and stability in murine models. An optimal dose of 1.85 μg per mouse was determined to evoke strong and lasting antibody responses against all 14 HPV types. This vaccine candidate also showed consistent potency across different production batches and proved to be stable under various storage conditions. Importantly, the significant correlation observed between pseudovirion-based neutralization assays (PBNAs) and total IgG Luminex immunoassays (LIAs) in multiple studies underscores the utility of the Luminex-based total IgG approach as an alternative for assessing immunogenicity. Notably, Sinocelltech Ltd.‘s total IgG LIA assay has progressed from preclinical evaluations to testing human serum samples in phase I/II/III clinical trials. This simplified assay method can effectively predict neutralization responses and has great potential to accelerate the development of HPV vaccines in line with the WHO’s ambitious cervical cancer elimination goal of vaccinating 90% of adolescent girls worldwide by 2030.

## Figures and Tables

**Table 1:** Advantages and disadvantages of PBNA and total IgG LIA.

Detection Method	Time to Results	Advantages	Disadvantages
PBNA	3–4 d	Directly measures the mechanism-based biological activity of the antibodies induced by the HPV vaccine. Detects all immunoglobin classes (IgM, IgA, and IgG)	Involves intricate procedures Demands significant labor and time investment Labor-intensive, relatively higher variable, time-consuming, low-throughput, and costly Slight cross-reactions between HPV6 and HPV11
Total IgG LIA	1 d	Detects all IgG binding to the HPV VLP, encompassing all epitopes with neutralizing potential High-throughput capability: Luminex enables simultaneous detection of up to 100 targets (e.g., 14 HPV VLP targets in this paper) per well in a single reaction. It employs color-coded beads tailored for specific targets, enabling the simultaneous detection of multiple analytes in a single reaction. This approach conserves samples, reagents, and detection time, making it particularly advantageous for clinical applications involving limited or precious samples. User-friendly operation, high sensitivity, reproducibility, fast turnaround time, and cost-effectiveness. Minimal cross-reaction observed across various vaccines types	Requires the use of costly color-coded magnetic beads May detect non-neutralizing antibodies

PBNA, pseudovirion-based neutralization assay; total IgG LIA, total IgG Luminex immunoassay.

**Table 2:** Antigen composition for the 14 HPV types.

Antigen Content for HPV Types 6/11/16/18/31/33/35/39/45/51/52/56/58/59 (μg/0.5 mL)	Total Antigen (μg)
30/40/60/40/20/20/20/20/20/20/20/20/20/20	370

**Table 3:** Vaccination procedures and sample collection details.

Study	Assay	Number of Animals	Dose (μg)	Dilution	Dosing Frequency (Day)	Serum Collection Timepoint (Day)
1#	Assay 1	10	0.93	0.5×	D0, D7, D21	D28
	Assay 1, 2	10, 10	1.85	1×	D0, D7, D21	D28
	Assay 1, 2	10, 10	2.31	1.25×	D0, D7, D21	D28
	Assay 1	10	3.70	2×	D0, D7, D21	D28
2#	From assay 1	10	1.85	1×	D0, D7, D21	D28
	Assay 3	30	1.85	1×	D0, D7, D21	D28
	Assay 4	10	1.85	1×	D0, D7, D21	D28
	Assay 5	10	1.85	1×	D0, D7, D21	D28
3#	From assay 3	10	1.85	1×	D0, D7, D21	D28 (3rd 1 W)
	Assay 6	11	1.85	1×	D0, D7, D21	D35 (3rd 2 W)
	Assay 7	10	1.85	1×	D0, D7, D21	D56 (3rd 5 W)
	Assay 8	10	1.85	1×	D0, D7, D21	D63 (3rd 6 W)
	Assay 9	10	1.85	1×	D0, D7, D21	D105 (3rd 12 W)

**Table 4:** The temperatures and time points in mouse potency assays.

Temperature	Time Points
2~8 °C	0, 3 m, 6 m, 9 m, 12 m, 18 m, 24 m, 38 m
25 °C	0, 12 w, 24 w
37 °C	0, 4 w, 12 w

**Table 5:** Cutoff values of total IgG LIA for 14 HPV types.

HPV Type	6	11	16	18	31	33	35	39	45	51	52	56	58	59
Cutoff value (MFI)	200	296	113	138	226	149	134	169	156	116	166	183	158	328

**Table 6:** Correlation coefficients in individual studies and combined Study #1, Study #2, and Study #3, and analysis of correlation coefficient variation across these studies.

HPV Type	Study #1 (n = 60)		Study #2 (n = 60)		Study #3 (n = 50)		Combined (n = 150)		CV%
	r	*p* Value	r	*p* Value	r	*p* Value	r	*p* Value	
HPV6	0.60	<0.0001	0.59	<0.0001	0.79	<0.0001	0.56	<0.0001	17
HPV11	0.63	<0.0001	0.63	<0.0001	0.73	<0.0001	0.72	<0.0001	8
HPV16	0.82	<0.0001	0.62	<0.0001	0.60	<0.0001	0.68	<0.0001	18
HPV18	0.67	<0.0001	0.56	<0.0001	0.47	0.0006	0.55	<0.0001	18
HPV31	0.45	0.0003	0.59	<0.0001	0.67	<0.0001	0.71	<0.0001	20
HPV33	0.44	0.0004	0.69	<0.0001	0.57	<0.0001	0.60	<0.0001	22
HPV45	0.54	<0.0001	0.52	<0.0001	0.56	<0.0001	0.51	<0.0001	4
HPV52	0.76	<0.0001	0.79	<0.0001	0.71	<0.0001	0.73	<0.0001	6
HPV58	0.84	<0.0001	0.74	<0.0001	0.55	<0.0001	0.62	<0.0001	21
HPV35	0.80	<0.0001	0.75	<0.0001	0.73	<0.0001	0.76	<0.0001	4
HPV39	0.60	<0.0001	0.62	<0.0001	0.75	<0.0001	0.82	<0.0001	12
HPV51	0.44	0.0004	0.78	<0.0001	0.81	<0.0001	0.72	<0.0001	30
HPV56	0.41	0.0010	0.71	<0.0001	0.69	<0.0001	0.68	<0.0001	27
HPV59	0.51	<0.0001	0.78	<0.0001	0.89	<0.0001	0.88	<0.0001	27
Range	0.41–0.84	/	0.52–0.79	/	0.47–0.89	/	0.51–0.88	/	4–27

## Data Availability

The sponsor, investigator, and other collaborators will review and approve data sharing proposals directed to the corresponding author based on scientific merit.

## Supplementary Materials

The following supporting information can be downloaded at: https://www.mdpi.com/article/10.3390/vaccines12111262/s1, Table S1. Time Schedule for Doses Administration in Mice and Serological Testing. Table S2. The Doses of Mouse Potency and Mean ED_50_ of Three Batches of 14-valent Vaccine. Table S3. Robustness Results of PBNA at Different GFP Expressing Levels of Pseudovirions. Table S4. The PBNA GMT Titers and Total IgG LIA GMT MFI Results in Study 2#. Table S5. Comparison of Correlation Coefficients of MFI-based and Unit-based (mNU/mL) Measurements between PBNA and Total IgG LIA in Cynomolgus Monkey Model [45].
