# Supplementary material for: Immunogenicity Assessment of a 14-Valent Human Papillomavirus Vaccine Candidate in Mice
Source: Vaccines (Basel). 2024 Nov 8;12(11):1262. doi: 10.3390/vaccines12111262 (PMC11599024; doi:10.3390/vaccines12111262)
Supplement: Supplementary file 1 [file vaccines-12-01262-s001.zip › vaccines-3260730-supplementary.pdf]

Supplemental Information for

Immunogenicity Assessment of A 14-Valent Human Papillomavirus Vaccine Candidate in Mouse

Lei Bei[1,†], Shuman Gao[2,†], Dandan Zhao[2], Yajuan Kou[2], Siyu Liang[2], Yurong Wu[2], Xiao Zhang[2], Dan Meng[2], Jianbo Lu[2], Chunxia Luo[2], Xuefeng Li[2], Yang Wang[2], Hongbin Qiu[1] and Liangzhi Xie[2,3,4]*

[1]Basic medical college of Jiamusi University, Heilongjiang, 154007, China
[2]Beijing Engineering Research Center of Protein and Antibody, Sinocelltech Ltd., Beijing 100176, China
[3]Beijing Key Laboratory of Monoclonal Antibody Research and Development, Sino Biological Inc., Beijing 100176, China
[4]Cell Culture Engineering Center, Chinese Academy of Medical Sciences & Peking Union Medical College, Beijing 100005, China

***Correspondence:**

Liangzhi Xie LX@sinocelltech.com

† Lei Bei and Shuman Gao contributed equally to this work.

This document file includes:

1 Methods

1.1 Quality Confirmation of HPV VLP

1.2 Animal Ethics

1.3 Validation of PBNA

1.4 Validation of Total IgG LIA

2 Tables S1 to S5

Table S1 Time Schedule for Doses Administration in Mice and Serological Testing

Table S2 The Doses of Mouse Potency and Mean ED_{50} of Three Batches of 14-valent Vaccine

Table S3 Robustness Results of PBNA at Different GFP Expressing Levels of Pseudovirions

Table S4 The PBNA GMT Titers and Total IgG LIA GMT MFI Results in Study 2#

Table S5 Comparison of Correlation Coefficients of MFI-based and Unit-based (mNU/mL) Measurements between PBNA and Total IgG LIA in Cynomolgus Monkey Model

1 Methods

1.1 Quality Confirmation of HPV VLP

The structural integrity of the individual HPV drug substance was confirmed by multiple analytical methods including dynamic light scattering (DLS), tungsten-stained transmission electron microscope (TEM), and differential centrifugal sedimentation (DCS) analysis. DLS, which measures particle size integrity by measuring fluctuation of scattered light intensity over time, showed that the protein particle size and its distribution met the quality specifications for each batch of drug substance (about 60 nm in diameter). TEM evaluated the particle conformation and morphology of each VLP type and showed particle uniformity and size consistency, consistent with the diameter results from DLS (data now shown). DCS was performed using a CPS disc centrifugal nanoparticle size analyzer, which is considered a reliable instrument for particle size distribution. It provided reliable particle size distribution information (data now shown), which is consistent with TEM results, indicating uniform particle size distribution. Results from these analyses confirmed the structural integrity of the HPV drug substances.

Product purity assessment included size exclusion chromatography (SEC-HPLC), SDS-PAGE, host protein residues and exogenous DNA contents analysis. SEC-HPLC evaluated purity by gel chromatography column separation, showing that the purity of VLP was above 95%. SDS-PAGE showed that the protein purity and the percentage of intact protein monomers were above 99.0% and 90.0%, respectively. The host protein residues (including HighFive cell genomic DNA and SF9 cell genomic DNA), which are indicaters of safety and purity, were detected by double antibody sandwich ELISA (DAS-ELISA) and were less than 0.5 ng/dose. The exogenous DNA content was evaluated by Taqman fluorescent probe qPCR, and the residual amout was less than 0.005 ng/dose.

To confirm the type specificity of VLPs, we used 14 type-specific neutralizing monoclonal antibodies (developed by Sinocell Ltd.) for ELISA detection before immunization and PBNA detection after mouse immunization [1]. The ELISA results

confirmed the type-specific antibodies of each batch of 14-valent VLPs [35]. The PBNA test results showed that there was no obvious cross-neutralization between the sera of different mice sera immunized with a single type VLP, and there was a slight cross-reaction between HPV6 and HPV11, which is consistent with the results of the previous studies [35, 45].

1.2 Animal Ethics

The anmimal studies were approved by the Ethics Review Committee on Welfare of Laboratory Animals at the National Institute for Food and Drug Control, with the ethics approval code "Zhongjian Dong No. 2018 (B) 020." Approval was granted in December 2018.

1.3 Validation of PBNA

The validation of the PBNA method was conducted in accordance with stringent criteria outlined in the guidelines and recommendations for the bioanalytical method validation. To thoroughly validate the PBNA method, we prepared a 14-valent HPV antibody samples (14v mixtures) containing 14 type-specific neutralizing monoclonal antibodies. A comprehensive validation process was undertaken, including specificity, accuracy, precision, and robustness of the PBNA method.

In the precision validation phase, the relative standard deviation (RSD) of 14v mixtures and HPV16, 18 international standards ranged from 10% to 40% across different days and technicians, adhering to the criteria of RSD≤50%. The results demonstrated the good precision of the method. In the accurate validation assay, the ratio of expected titer to detected titer values for 1-, 3-, and 9-fold diluted 14v samples neutralizing 14 pseudovirions fell within the range of 0.7 to 1.5, meeting the acceptance criteria of 0.3 to 3.0. Further validation was carried out by combining the 14v samples with blank mice sera and combining the 14v samples with HPV16/18 international standards at twice the International Unit of HPV16/18 international standards (data not shown), confirming the accuracy characteristics of the method.

The method's robustness was established by assessing its performance under varying conditions. Cell density within the range of 12,000 to 20,000 cells/wells (RSD: 0-30%) and incubation time between 60 and 96 hours (RSD: 0-20%) exhibited no significant impact on neutralization titers, adhering to the criteria of RSD≤50%. Additionally, the GFP points of pseudovirions were optimized, and neutralization titers of 14v mixtures were determined by incubating them with diluted pseudovirions at different GFP points ranging from 100 to 1500. The results demonstrated that when the GFP points of 14 pseudovirions fell within a specific range meeting the quality

standard (RSD < 50%), no substantial effect on the neutralization titers was observed (Table S3).

The specificity of method was evaluated by determining the EC_{50} of immunized serum using single type VLPs against 14 types of pseudovirions separately. The data demonstrated the specificity of the method, indicating minimal cross-reaction among various types of VLPs. A slight cross-reaction was observed solely between HPV 6 and HPV 11 [1].

1.4 Validation of Total IgG LIA

In the mice model, a challenge of obtaining sufficient mice standard serum and blank serum for validation arises due to the limited serum volume that can be collected from mice, considering their small size. Consequently, this limitation prevents the comprehensive validation of total IgG method in the murine model. However, we have successfully conducted a comprehensive validation of the Luminex-based total IgG assay using cynomolgus monkey model [35] and human clinical trial serum matrices [39]. Both sets of data have confirmed the robustness and accuracy of total IgG method, thereby providing strong evidence for its suitability in evaluating immunogenicity across diverse serum matrices.

2 Table

Table S1 Time Schedule for Doses Administration in Mice and Serological Testing

Study	Assay	Number of animals	Dose (μg)	Dilutions	Dosing frequency (day)	Serum collection timepoint (day)	Time of the first dose	Time of the second dose	Time of the third dose	Serum collection time	PBNA detection time	Total IgG detection time
1#	Assay 1	10	0.93	0.5×	D0, D7, D21	D28	2020.07.14	2020.07.21	2020.08.04	2020.08.11	2020.08.21-2020.09.08	2020.10.28
		10	1.85	1×	D0, D7, D21	D28						
		10	2.31	1.25×	D0, D7, D21	D28						
		10	3.7	2×	D0, D7, D21	D28						
	Assay 2	10	1.85	1×	D0, D7, D21	D28	2020.10.01	2020.10.08	2020.10.22	2020.10.29	2020.11.02-2020.12.25	2020.11.04
		10	2.31	1.25×	D0, D7, D21	D28						
2#	From assay 1	10	1.85	1×	D0, D7, D21	D28	2020.07.14	2020.07.21	2020.08.04	2020.08.11	2020.08.21-2020.09.08	2020.10.28
	Assay 3	30	1.85	1×	D0, D7, D21	D28	2020.06.26	2020.07.03	2020.07.17	2020.07.24	2020.07.27-2020.09.02	2020.10.27
	Assay 4	10	1.85	1×	D0, D7, D21	D28	2020.07.15	2020.07.22	2020.08.05	2020.08.12	2020.08.31-2020.09.29	2020.10.27
	Assay 5	10	1.85	1×	D0, D7, D21	D28	2020.08.17	2020.08.24	2020.09.07	2020.09.14	2020.09.21-2020.10.05	2020.10.28
3#	From assay 3	10	1.85	1×	D0, D7, D21	D28 (3rd 1W)	2020.06.26	2020.07.03	2020.07.17	2020.07.24	2020.07.27-2020.09.02	2020.10.27
	Assay 6	11	1.85	1×	D0, D7, D21	D35 (3rd 2W)				2020.07.31	2020.08.11-2020.11.18	2020.11.18

Assay 7	10	1.85	1×	D0, D7, D21	D56 (3rd 5W)	2020.08.21	2020.10.30-2020.11.24	2020.11.18
Assay 8	10	1.85	1×	D0, D7, D21	D63 (3rd 6W)	2020.08.28	2020.11.06-2020.11.24	2020.11.18
Assay 9	10	1.85	1×	D0, D7, D21	D105 (3rd 12W)	2020.10.09	2020.11.09-2020.11.18	2020.11.18

Table S2 The Doses of Mouse Potency and Mean ED_{50} of Three Batches of 14-valent Vaccine

Types	Doses (μg)	Mean ED_{50} (μg) ± SEM
HPV6	0.0002, 0.0006, 0.0019, 0.0056, 0.0167, 0.05, 0.15	0.0023±0.0003
HPV11	0.0003, 0.0008, 0.0025, 0.0074, 0.0222, 0.0667, 0.20	0.0017±0.0001
HPV16	0.0004, 0.0012, 0.0037, 0.0111, 0.0333, 0.1000, 0.30	0.0007±0.0001
HPV18	0.0003, 0.0008, 0.0025, 0.0074, 0.0222, 0.0667, 0.20	0.0006±0.0001
HPV31	0.00014, 0.00041, 0.00123, 0.0037, 0.01, 0.03, 0.1	0.0005±0.0001
HPV33		0.0007±0.0003
HPV45		0.0048±0.0022
HPV52		0.001±0.0003
HPV58		0.0002±0.0001
HPV35		0.0023±0.0003
HPV39		0.0002±0
HPV51		0.0141±0.0026
HPV56		0.0009±0.0002
HPV59		0.0041±0.0004

Table S3 Robustness Results of PBNA at Different GFP Expressing Levels of Pseudovirions

Pseudovirion type	GFP points range	GFP points	EC_{50}	Geometric mean titer (GMT)	RSD（%）
HPV6	118-1526	118	6687	10847	50
		222	7029		
		478	18396		
		811	15655		
		1265	14567		
		1526	8262		
HPV11	171-2354	171	8964	19630	50
		348	11147		
		741	31267		
		1355	27695		
		1804	27110		
		2354	24392		
HPV16	68-938	68	27510	24163	40
		175	38650		
		469	27599		
		938	23518		
HPV18	80-1188	80	26278	40990	50
		182	82920		
		332	43816		
		525	44008		
		847	46011		
		1188	24535		
HPV31	78-852	78	18300	15107	40
		142	24541		
		284	18505		
		533	12459		
		852	7599		
HPV33	73-1573	73	36957	28715	20
		128	28459		
		315	26641		
		523	35280		
		891	29383		
		1573	19299		
HPV35	60-1257	60	14573	21157	40
		123	35942		
		272	26246		
		510	27975		
		829	21422		

Pseudovirion type	GFP points range	GFP points	EC_{50}	Geometric mean titer (GMT)	RSD（%）
		1257	10886		
HPV39	66-1507	66	8952	11639	20
		141	10579		
		308	16567		
		538	12711		
		912	11215		
		1507	11113		
HPV45	79-1619	79	36137	22039	30
		99	18706		
		262	20957		
		584	24216		
		1039	21415		
		1619	15600		
HPV51	57-1331	57	50118	33754	40
		125	53908		
		232	38935		
		430	39292		
		721	22393		
		1331	15979		
HPV52	153-507	153	14866	7959	50
		223	9963		
		301	6649		
		431	6261		
		507	5179		
HPV56	98-1600	98	19985	14419	50
		153	11928		
		298	14765		
		658	26254		
		911	10504		
		1600	9257		
HPV58	109-1953	109	18164	20196	30
		179	12131		
		398	19996		
		734	28681		
		1227	28238		
		1953	19019		
HPV59	47-1708	47	12939	14601	20
		152	14749		
		354	11822		
		824	18551		

Pseudovirion type	GFP points range	GFP points	EC_{50}	Geometric mean titer (GMT)	RSD（%）
		1708	15858		

Table S4 The PBNA GMT Titers and Total IgG LIA GMT MFI Results in Study 2#

Types	PBNA				Total IgG LIA GMT			
	GMT Titer	Standard Error (SE)	Cutoff	GMT: Cutoff	GMT MFI	Standard Error (SE)	Cutoff	GMT: Cutoff
HPV6	3924	2825	80	49	2308	252	200	12
HPV11	4959	1649	80	62	4125	398	296	14
HPV16	7121	1353	80	89	9437	751	113	84
HPV18	5393	789	80	67	4909	441	138	36
HPV31	5724	811	80	72	2885	332	226	13
HPV33	2239	1425	80	28	2454	420	149	16
HPV45	1472	278	80	18	1590	238	166	10
HPV52	4893	664	80	61	2990	388	158	19
HPV58	10693	2131	80	134	5882	564	134	44
HPV35	1747	507	80	22	1128	255	169	7
HPV39	8490	891	80	106	3414	266	156	22
HPV51	3146	730	80	39	4101	592	116	35
HPV56	3309	740	80	41	1547	403	183	8
HPV59	919	250	80	11	1121	151	328	3

Table S5 Comparison of Correlation Coefficients of MFI-based and Unit-based (mNU/mL) Measurements between PBNA and Total IgG LIA in Cynomolgus Monkey Model

HPV type	Correlation coefficient (r)		CV%
	MFI	Unitage concentration (mNU/mL)	
HPV6	0.94	0.95	0%
HPV11	0.80	0.76	3%
HPV16	0.81	0.83	1%
HPV18	0.86	0.87	0%
HPV31	0.87	0.86	1%
HPV33	0.97	0.97	0%
HPV35	0.88	0.85	2%
HPV39	0.95	0.95	0%
HPV45	0.83	0.78	3%
HPV51	0.82	0.75	5%
HPV52	0.66	0.63	2%
HPV56	0.83	0.77	4%
HPV58	0.71	0.64	6%
HPV59	0.79	0.73	5%

To compare the correlation coefficients of MFI-based and unit-based (mNU/mL) measurements between PBNA and Total IgG LIA, we analyzed data obtained from cynomolgus monkeys diluted 4000 times, closely approximating the 5000-fold dilution used in the mouse model. Serum samples were collected on D31 (prior to the second dose), D87 (eight weeks after the second dose), and D91 (prior to the third dose) as reported in Bei et al., 2022 [35]. The dataset included 31-36 data points per type, with some data from certain types adjusted for dilution during retesting excluded from the analysis. The 4000-fold dilution was selected to closely mimic the conditions of mouse serum dilution, ensuring a reliable comparison.
